# Prevalence of Overweight and Obesity Among Students of Government and Private High Schools in an Indian State With Significant Tribal Population: A Cross-Sectional Analytical Study

**DOI:** 10.7759/cureus.43105

**Published:** 2023-08-07

**Authors:** Syed Hedayetullah, Vidya Sagar, Shashi B Singh, Pradeep Minz, Anit Kujur, Vivek Kashyap, Md Sariful Haque, Dilip Kumar, Surendra Sahu, Apoorva Wasnik

**Affiliations:** 1 Community Medicine, Rajendra Institute of Medical Sciences, Ranchi, IND; 2 Preventive and Social Medicine, Rajendra Institute of Medical Sciences, Ranchi, IND; 3 Physiology, Rajendra Institute of Medical Sciences, Ranchi, IND

**Keywords:** tribal, school children, prevalence, obesity, overweight, cross-sectional analytical study

## Abstract

Background: Overweight and obesity among school-going children is an emerging public health problem in the country. The information available on the true extent of obesity and overweight among school-aged children is limited. Hence, the present study has been conducted to determine the prevalence of overweight and obesity among high school students in Jharkhand, India.

Methodology: This was a cross-sectional study conducted among 1162 students of government and private schools of Ormanjhi block, Ranchi district, from July 2022 to December 2022. A predesigned, semi-structured, pre-tested questionnaire containing different sections namely sociodemographic characteristics, and health parameters were used for the study subjects. Clinical examination and anthropometric measurements of height, weight, and waist and hip circumferences were taken using standard equipment to calculate body mass index (BMI) and central obesity (waist-hip ratio).

Results: The prevalence of overweight and obesity was more at 14 years of age (30.2%), among boys (18.1%), and among students practicing the Islam religion (51.1%). Moreover, the prevalence of overweight and obesity was found to be highest in private schools (66.2%), and that was statistically significant (p<0.001).

Conclusion: The prevalence of overweight and obesity was found to be significant with respect to age, gender, and religion. The findings from this study would be helpful in raising awareness among students, parents, teachers, and health professionals about the influence of overweight and obesity on a child’s physical, social, and psychological well-being, and this, in turn, would facilitate parents, students, and teachers in the adoption of a healthy lifestyle.

## Introduction

As we know that overweight and obesity among children are emerging public health problems in India. According to WHO, overweight and obesity are defined as conditions of abnormal or excessive fat accumulation that may impair health [1]. In fact, obesity is considered a common nutritional disorder and a major public health hurdle. It is caused as a result of abnormal growth of the adipose tissue either due to enlargement of fat cell size (hypertrophic obesity), an increase in the number of fat cells (hyperplastic obesity), or a combination of both [2]. Obesity is one of the most prevalent forms of malnutrition, common in both children as well as adults [3]. It is also common in both developing and developed countries. The prevalence of obesity has tripled since 1975.

Childhood obesity is one of the serious public health predicaments of the 21st century. The proportion of overweight and obesity is greater in urban areas at 33.2% in comparison to 19.7% in rural areas [4]. Overweight and obesity were earlier considered global public health problems of high-income and developed countries; however, they are now rising even in low- and middle-income developing countries [5]. It has been estimated that there is an increased heterogeneity in the prevalence of overweight and obesity with implications for global actions and targets across the world [6]. Among school-going children, there has been a 10-fold increase in the prevalence of obesity from 1990 to 2015, and the International Association for the Study of Obesity (IASO) and International Obesity Task Force (IOTF) reckon that 200 million school children across the globe are either overweight or obese [7]. The increasing trend of childhood overweight and obesity has been seen all over India. A systematic review conducted by Gupta et al. reported that the prevalence of overweight in the age group of 5-19 years ranged between 6.1% and 25.2%, while obesity was found to be between 3.6% and 11.7% [8], which is classified as per WHO classification of nutrition [2,3].

Although childhood obesity is one of the most serious public health predicament of the 21st century, information available on the true extent of obesity and overweight among school-aged children are limited. Hence, the present study has been conducted to determine the burden of overweight and obesity among school-going children in a tribal state in eastern India. To the best of our knowledge, this is the first such study from this area.

## Materials and methods

This was a cross-sectional analytical study conducted among 1162 students of government and private schools of Ormanjhi block, Ranchi, Jharkhand, India, from July 2022 to December 2022. The data was analyzed in the Department of Community Medicine of Rajendra Institute of Medical Sciences, Ranchi. Approval of the Institutional Ethics Committee, Rajendra Institute of Medical Sciences was taken prior to the study vide memo no 325 dated July 27, 2021 . The sample size was calculated as 1162 by considering the prevalence of childhood overweight and obesity as 11.7% based on a previous study [9], with a precision of 2% and a dropout rate of 10%.

For the data collection procedure, first of all, a list of government and private high schools was obtained from the office of the Block Education Officer (BEO), Ormanjhi, Ranchi, Jharkhand. Then three government (out of four) and four private recognized (out of six) high schools were selected. Necessary permission from the concerned authority including the BEO was obtained before the start of the study. In order to ensure complete coverage, two visits were made to each school. Students of government and private high schools of Ormanjhi block, Ranchi, who were present at the school during both or either of these two visits were included. Exclusion criteria were: children who were not present in the school during both the two visits. Initially, meetings were conducted with principals and teachers of these schools to explain the purpose of the study, and consent was obtained from the participants before enrolling them in the study. Then the students were selected according to their probability proportion to size. A predesigned, semi-structured, pre-tested questionnaire containing sections on sociodemographic characteristics and health parameters were used for the study subjects. Clinical examination and anthropometric measurements of height, weight, and waist and hip circumference were taken using standard equipment like a stadiometer, bathroom scale weighing machine, and non-stretchable measuring tape, respectively, to calculate BMI (Table 1).

**Table 1:** WHO classification of nutrition conditions in children and adolescents based on anthropometry Age: 61 months to 19 years

Classification based on BMI	Indicator and cut-off
Overweight	BMI-for-age >1 SD (equivalent to BMI 25 kg/m^2^ at 19 years)
Obese	BMI-for-age >2 SD (equivalent to BMI 30 kg/m^2^ at 19 years)
Thin	BMI-for-age < –2 to –3 SD
Severely thin	BMI-for-age

## Results

Out of 1162 study participants, a total of 1110 students completed the questionnaire with a response rate of 95.52%. The age and gender-wise distribution are shown in Table 2. Out of 1110 study participants who completed the questionnaire in our study, 580 (52.25%) were girls, and 530 (47.74%) were boys. The majority of the study participants (30.70%) were aged 14 years, followed by children aged 15 years (27.8%) and 13 years (16.9%). The overall mean age of study subjects was 14.46 + 1.29 years with a range from 11 to 19 years. In this study, the highest prevalence of overweight/obesity was found in children aged 14 years of age (30.2%), followed by 22.3% in children aged 16 years or more. The difference in the proportion of overweight/obesity in different age groups was found to be statistically significant (p < 0.001) (Table 3).

**Table 2:** Showing age and gender-wise distribution of the study subjects

Age (years)	Boys	Girls	Total
11	4 (0.8%)	2 (0.3%)	6 (0.5%)
12	22 (4.2%)	31 (5.3%)	53 (4.8%)
13	71 (13.4%)	117 (20.2%)	188 (16.9%)
14	154 (29.1%)	187 (32.2%)	341 (30.7%)
15	149 (28.1%)	160 (27.6%)	309 (27.8%)
>16	130 (24.5%)	83 (14.3%)	213 (19.2%)
Total	530 (100.0%)	580 (100.0%)	1110 (100.0%)

**Table 3:** Prevalence of overweight/obesity according to age X^2^=79.983; p-value <0.001 (significant)

Age in years	Normal	Overweight/Obesity	Underweight	Total
11	3 (0.3%)	3 (2.2%)	0 (0.0%)	6 (0.5%)
12	25 (2.9%)	24 (17.3%)	4 (3.6%)	53 (4.8%)
13	141 (16.4%)	21 (15.1%)	26 (23.4%)	188 (16.9%)
14	265 (30.8%)	42 (30.2%)	34 (30.6%)	341 (30.7%)
15	267 (31.0%)	18 (12.9%)	24 (21.6%)	309 (27.8%)
>16	159 (18.5%)	31 (22.3%)	23 (20.7%)	213 (19.2%)
Total	860 (100.0%)	139 (100.0%)	111 (100%)	1110 (100.0%)

**Figure 1:**
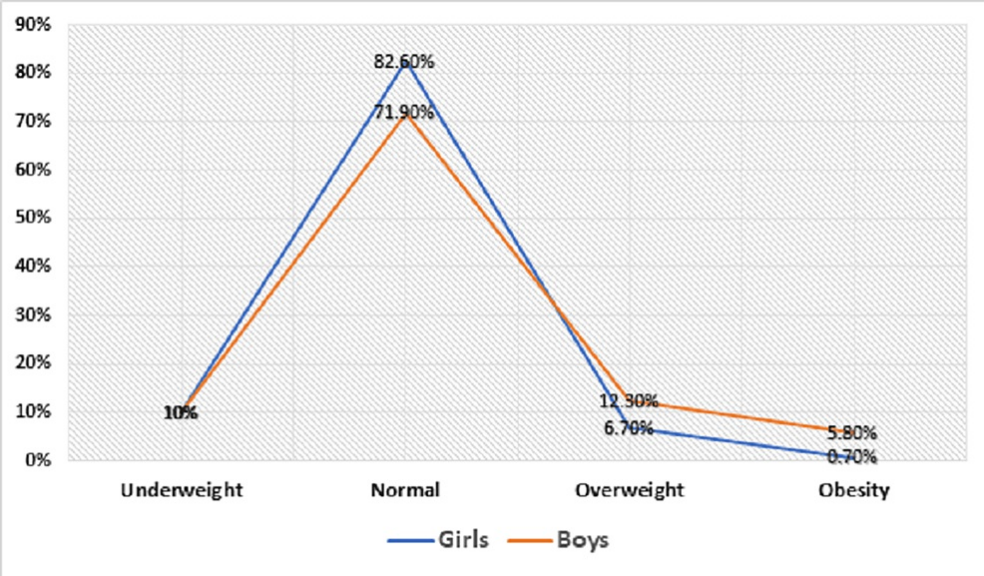
Prevalence of overweight and obesity according to gender

Out of 1110 study participants, 39 (6.7%) of 580 girls were overweight and four girls (0.7%) were obese, while 65 (12.3%) of 530 boys were overweight and 31 boys (5.8%) were obese. A statistically significant association was found between sex and prevalence of overweight/obesity (p < 0.001) (Figure 1).

In the context of religion, out of 1110 study participants, 628 (56.6%) were Hindus, of which 54 (38.8%) were overweight/obese. The prevalence of overweight/obesity was highest in Muslims (n=71; 51.1%) followed by Hindus (38.8%) and by others like Christians and Sarna (10.2%). The difference in the prevalence of overweight/obesity with respect to religion was found to be statistically significant (p = 0.001) (Table 4).

**Table 4:** Prevalence of overweight/obesity according to religion Pearson Chi-Square X^2^ = 27.271; p-value <0.001 (significant)

Religion	Normal	Overweight/obesity	Underweight	Total
Hindu	512 (59.5%)	54 (38.8%)	62 (55.9%)	628 (56.6%)
Muslim	257 (29.9%)	71 (51.1%)	34 (30.6%)	362 (32.6%)
Christian	16 (1.9%)	3 (2.2%)	2 (1.8%)	21 (1.9%)
Sikh	1 (0.1%)	0 (0.0%)	0 (0.0%)	1 (0.1%)
Sarna	74 (8.6%)	11 (7.9%)	13 (11.7%)	98 (8.8%)
Total	860 (100%)	139 (100%)	111 (100%)	1110 (100%)

**Figure 2:**
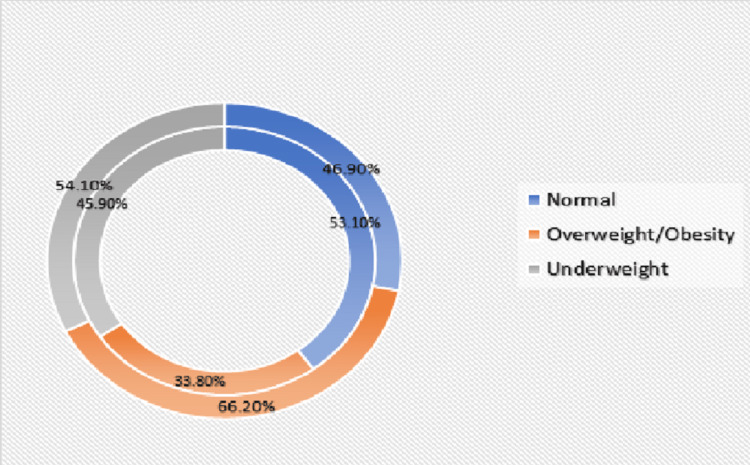
Comparison in the prevalence of overweight/obesity between government and private schools

The prevalence of overweight/obesity was high in private schools (66.2%) whereas 33.8% of the study participants were overweight/obese in government schools. There was a statistically significant difference (p < 0.001) in the prevalence of overweight/obesity with respect to type of school (Figure 2).

## Discussion

Overweight and obesity among school-going children has been emerging as an important public health hazard in most parts of the countries of the world including India. Although studies are being conducted in various regions of our country and they clearly indicate that childhood obesity is quite alarming, most of the studies conducted till now in our country are in urban schools and the trend in rural areas has not been studied in detail so far. Hence, an attempt was made to study the prevalence of childhood overweight/obesity in the government and private high schools of Ormanjhi block of Ranchi district in India, which belongs to the rural area.

Out of 1110 study participants in our study, 580 (52.25%) were girls and 530 (47.74%) were boys. In the present study, the highest prevalence of overweight/obesity was found in children aged 14 years (30.2%), followed by 22.3% who were aged 16 years or more. The difference in the proportion of overweight/obesity in different age groups was found to be statistically significant (p < 0.001). In a study done by Mohanty in Puducherry, the prevalence of overweight (5.2%) and obesity (3.89%) was found to be highest in children aged 15 years and a positive correlation was found between age and obesity [10]. On the other hand, prevalence was found to be higher in children aged <15 years in a study done by Bharati et al. [11]. The findings in a study done by Chaatwal showed that the prevalence of obesity decreased significantly with age, from 18.5% at nine years to 7.6% at 14 years, rising at 15 years to 12.1% [12]. This might be due to the fact that fat tissue and overall body weight increase in children during puberty; the number of fat cells increases during periods of rapid growth up to 16 years of age, after which increased fat ordinarily accumulates by increasing the size of the fat cells already present [13,14]. In our study, the prevalence of overweight/obesity at 11 years of age was found to be 0.27. Whereas, prevalence was 2.16%, 1.89%, 3.78%, 1.62%, and 2.79% at 12 years, 13 years, 14 years, 15 years, and 16 years of age group respectively.

In the current study, the prevalence of overweight and obesity was found to be more in boys (12.3% and 5.8%, respectively) as compared to that in girls (6.7% and 0.7%, respectively) and this was statistically significant. A similar finding was reported from studies done in Dakshina Kannada and Udupi districts by Kumar et al. [15] and in urban India by Ramachandran et al. [16], where it was observed that the prevalence of overweight and obesity was higher in boys compared to girls. In contrast to the above findings, however, in a study done in Puducherry by Mahajan et al. [17] and one in Saudi Arabia by El-Hazmi and Warsy [18], the prevalence of overweight and obesity was higher in girls compared to boys. In our study, the prevalence of overweight and obesity was more in boys as compared to that in girls. This might be becausefemale children tend to report liking and eating more foods that are lower in energy density and higher in critical nutrients (i.e., fruits and vegetables) than males [19]**.**

In our study, in the context of religion, the prevalence of overweight/obesity was high in students practicing Islam (51.1%) followed by those following Hinduism (38.8%) and other religions like Christianity and Sarna (10.1%). The difference in the prevalence of overweight/obesity with respect to religion was found to be statistically significant (p = 0.001). In contrast to our study, in a study done by Bharati et al., the prevalence of overweight/obesity was higher in school-going Hindu children in Wardha city [11].

It has been clear from the findings in our study that the prevalence of overweight/obesity was higher in private schools (66.2%). This is probably due to the fact that the majority of students studying in private schools belong to higher socioeconomic status. Comparatively, only 33.8% of the study participants studying in government schools were overweight/obese. There was a statistically significant difference in the prevalence of overweight/obesity with respect to type of school (p < 0.001). Our observations showed similar results to the results of previous studies [13].

Limitations of the study

This study has demonstrated the demographic characteristics as well as the prevalence of overweight and obesity in terms of age, gender, religion, and types of schools; however, various other predictors like physical activity and family history, which are also major contributors to overweight and obesity, were not considered in our study.

## Conclusions

The prevalence of overweight and obesity was found to be significant with respect to age, gender, and religion, where it was higher at 14 years of age, among males, and among those who practised the Islam religion. Moreover, the prevalence of overweight and obesity was found to be highest in private schools. These differences were statistically significant. The findings from this study would be helpful in raising awareness among students, parents, teachers, and health professionals about the influence of overweight and obesity on a child’s physical, social, and psychological well-being. The study would facilitate parents, students, and teachers in the adoption of a healthy lifestyle.
